# Hypertension urgencies in the SPYRAL HTN-OFF MED Pivotal trial

**DOI:** 10.1007/s00392-022-02064-5

**Published:** 2022-07-19

**Authors:** Michael A. Weber, Roland E. Schmieder, David E. Kandzari, Raymond R. Townsend, Felix Mahfoud, Konstantinos Tsioufis, Kazuomi Kario, Stuart Pocock, Fotis Tatakis, Sebastian Ewen, James W. Choi, Cara East, David P. Lee, Adrian Ma, Debbie L. Cohen, Robert Wilensky, Chandan M. Devireddy, Janice P. Lea, Axel Schmid, Martin Fahy, Michael Böhm

**Affiliations:** 1 Professor of Medicine, Division of Cardiovascular Medicine, Downstate Medical Center, SUNY Downstate College of Medicine, State University of New York, Brooklyn, NY USA; 2 University Hospital Erlangen, Erlangen, Germany; 3 Piedmont Heart Institute, Atlanta, GA USA; 4 Perelman School of Medicine, University of Pennsylvania, Philadelphia, PA USA; 5 Klinik Für Innere Medizin III, Universitätsklinikum Des Saarlandes, Saarland University, HomburgSaar, Germany; 6 Hippocratio Hospital, National and Kapodistrian University of Athens, Athens, Greece; 7 School of Medicine, Jichi Medical University, Tochigi, Japan; 8 London School of Hygiene and Tropical Medicine, London, UK; 9 Baylor Research Institute, Jack and Jane Hamilton Heart and Vascular Hospital, Dallas, TX USA; 10 Stanford Hospital and Clinics, Stanford, CA USA; 11 School of Medicine, Emory University, Atlanta, GA USA; 12 Medtronic PLC, Santa Rosa, CA USA

**Keywords:** Hypertension, Blood pressure, Renal denervation, Hypertensive urgency

## Abstract

The SPYRAL HTN-OFF MED Pivotal trial (https://clinicaltrials.gov/ct2/show/NCT02439749) demonstrated significant reductions in blood pressure (BP) after renal denervation (RDN) compared to sham control in the absence of anti-hypertensive medications. Prior to the 3-month primary endpoint, medications were immediately reinstated for patients who met escape criteria defined as office systolic BP (SBP) ≥ 180 mmHg or other safety concerns. Our objective was to compare the rate of hypertensive urgencies in RDN vs. sham control patients. Patients were enrolled with office SBP ≥ 150 and < 180 mmHg, office diastolic BP (DBP) ≥ 90 mmHg and mean 24 h SBP ≥ 140 and < 170 mmHg. Patients had been required to discontinue any anti-hypertensive medications and were randomized 1:1 to RDN or sham control. In this post-hoc analysis, cumulative incidence curves with Kaplan–Meier estimates of rate of patients meeting escape criteria were generated for RDN and sham control patients. There were 16 RDN (9.6%) and 28 sham control patients (17.0%) who met escape criteria between baseline and 3 months. There was a significantly higher rate of sham control patients meeting escape criteria compared to RDN for all escape patients (*p* = 0.032), as well as for patients with a hypertensive urgency with office SBP ≥ 180 mmHg (*p* = 0.046). Rate of escape was similar between RDN and sham control for patients without a measured BP exceeding 180 mmHg (*p* = 0.32). In the SPYRAL HTN-OFF MED Pivotal trial, RDN patients were less likely to experience hypertensive urgencies that required immediate use of anti-hypertensive medications compared to sham control.

**Graphical abstract:**

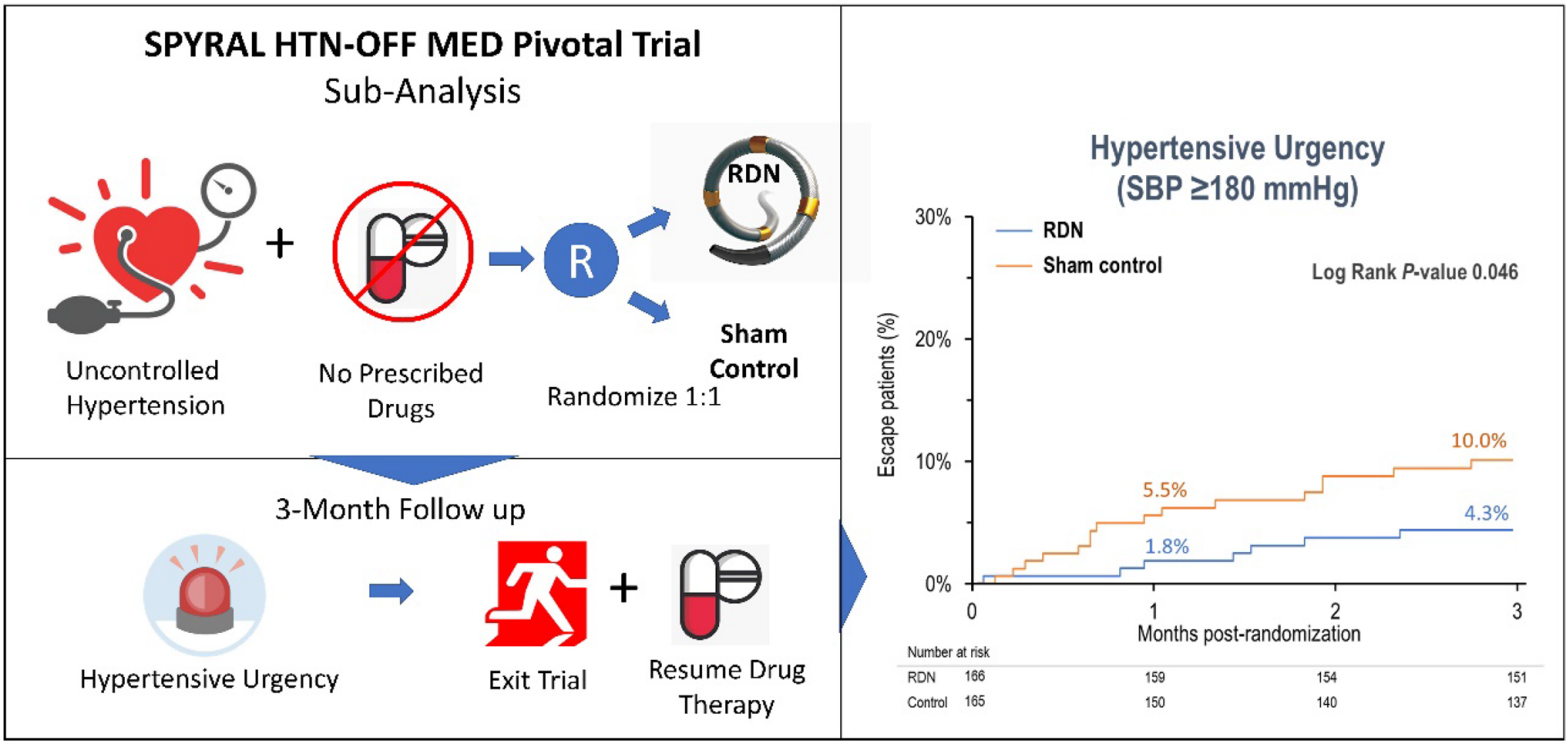

## Introduction

Over one third of adults are affected by hypertension, which is associated with an increased risk of cardiovascular events and stroke [1]. Recent ACC/AHA guidelines define a hypertensive emergency as systolic blood pressure (SBP) above 180 mmHg and/or diastolic blood pressure (DBP) above 120 mmHg [2]. Specifically, hypertensive urgencies are associated with severe blood pressure (BP) elevation in otherwise stable patients without acute or impending change in target organ damage or dysfunction [2]. Patients’ non-adherence to anti-hypertensive medications or inadequacy of these medications can lead to uncontrolled hypertension and hypertensive urgencies [3, 4], demonstrating the need for non-pharmacologic hypertension treatment options.

Results from randomized sham-controlled trials have shown the utility of renal denervation as an alternative or adjunctive option to pharmacologic therapy for hypertension [5, 6]. Primary results from the prospectively powered, sham-controlled SPYRAL HTN-OFF MED Pivotal trial demonstrated a reduction in 24 h systolic SBP after catheter-based renal denervation (RDN) at 3 months compared to sham control in the absence of anti-hypertensive medications [6]. Per protocol, patients who met escape criteria of office SBP ≥ 180 mmHg for hypertensive on urgency [2] or other safety concerns were able to resume anti-hypertensive medications at physician discretion. In this post-hoc analysis, we sought to examine the rate of patients meeting escape criteria in RDN and sham control groups in the SPYRAL HTN-OFF MED Pivotal trial.

## Methods

### Patients

The SPYRAL HTN-OFF MED Pivotal Trial is an international, prospective, single blinded, 1:1 randomized, sham-controlled trial, registered at ClinicalTrials.gov as NCT02439749. The design of the study and primary results have been previously reported [6, 7]. Briefly, patients were enrolled with typical uncontrolled hypertension defined as office SBP ≥ 150 mmHg and < 180 mmHg, office DBP ≥ 90 mmHg, and mean 24 h SBP ≥ 140 mmHg and < 170 mmHg using ambulatory BP monitoring. Patients were required to discontinue any anti-hypertensive medications 3–4 weeks prior to the planned procedure. Written informed consent was provided by all patients before enrollment. The trial protocol was approved by the institutional review board or ethics committee at each study site, and the trial was conducted in accordance with the Declaration of Helsinki.

### Procedures

After renal angiography revealed suitable renal anatomy, patients were randomized 1:1 to renal denervation or sham procedure [6, 7]. The Symplicity Spyral™ multi-electrode renal denervation catheter and the Symplicity G3™ radio-frequency generator (Medtronic, Minneapolis, MN, USA) were used for the RDN procedure. Sham control group patients remained on the table for at least 20 min after renal angiography to maintain blinding.

Office BP was measured at baseline and all follow-ups using an automatic BP monitor (Omron, Omron Healthcare, Inc, Lake Forest, IL, USA). Three seated BP measurements were obtained at least 1 min apart and averaged.

Patients who met escape criteria of office SBP ≥ 180 mmHg for hypertension urgency[2] or other safety concerns resumed anti-hypertensive medications as prescribed by their physicians. Escape patients discontinued the off-medications portion of the trial, but were still followed and included in the primary endpoint analysis. If a patient met escape criteria prior to measuring office and/or 24 h BP at 3 months, the last observation carried forward (measured 30 days or more past procedure up to and including escape date), was used for the primary endpoint analysis. Escape patients with no last observation carried forward were not included in the primary endpoint analysis.

### Statistical analysis

Continuous variables are presented as means ± standard deviation and compared between treatment arms using t-tests. Categorical variables are presented as counts and percentages and compared between treatment arms using Fisher’s exact test. Cumulative incidence curves with Kaplan–Meier estimates of rate of patients meeting escape criteria were generated and compared between treatment arms using log-rank tests. Statistical analyses were performed using SAS for Windows (version 9·4 or higher; SAS Institute, Cary, NC).

## Results

In the SPYRAL HTN-OFF MED Pivotal trial, there were 166 patients randomized to RDN and 165 randomized to sham control. Of these, there were 16 RDN patients (9.6%) compared to 28 sham control patients (17.0%) who met escape criteria between baseline and 3 months [6]. Of these patients, 7 RDN and 16 sham control patients had office SBP ≥ 180 mmHg. Other safety reasons for escape included headache, elevated BP (of concern to the investigator even if SBP not > 180 mmHg), or physician discretion. Mean BP at escape is detailed in Table 1.

**Table 1:** Mean office blood pressure measurements at time of escape

	RDN	Sham control
All patients meeting safety escape criteria	177/103 mmHg (*N* = 16)	176/108 mmHg (*N* = 27)
Escape due to SBP ≥ 180 mmHg (hypertensive urgency)	188/102 mmHg (*N* = 7)	187/108 mmHg (*N* = 15)
Escape due to other safety reason^1^	168/104 mmHg (*N* = 9)	161/108 mmHg (*N* = 12)

Office blood pressure measurements at time of escape not available for one patient

^1^Safety reasons included hypertension/hypertension crisis (5), headache (4), nausea and dizziness (1), blurry vision and worsening headache (1), fatigue (1), suspected transient ischemic attack (1) and physician discretion (8)

There were no significant differences in age, gender or race of escape vs. non-escape patients in either the RDN or sham control groups (Table 2), but diabetics appeared to have a higher chance of escape occurring than non-diabetics, especially in the control group. The overall population of type II diabetics in the RDN and sham control groups was similar (4% vs. 5%) [6]. Escape patients in the sham control group also had higher BMI and longer time since hypertension diagnosis compared to non-escape patients. Estimated glomerular filtration rate (eGFR) was similar between escape and non-escape patients in RDN and sham control groups.

**Table 2:** Baseline characteristics of escape vs. non-escape patients in RDN and sham control groups

	RDN			Sham control			Escape RDN vs. Sham *P*-value
	Escape (*N* = 16)	Non-Escape (*N* = 150)	*P*-value	Escape (*N* = 28)	Non-Escape (*N* = 137)	*P*-value	
Age	53.8 ± 13.6	52.3 ± 10.6	0.59	54.6 ± 10.2	52.1 ± 10.4	0.26	0.83
Male	50.0% (8/16)	66.0% (99/150)	0.27	75.0% (21/28)	67.2% (92/137)	0.51	0.11
BMI	30.3 ± 5.0	31.2 ± 6.1	0.56	32.8 ± 5.1	30.5 ± 5.5	0.038	0.12
Length of HTN diagnosis			0.35			0.024	0.30
0–5 years	43.8% (7/16)	44.7% (67/150)		28.6% (8/28)	48.9% (67/137)		
6–10 years	0.0% (0/16)	22.0% (33/150)		10.7% (3/28)	13.9% (19/137)		
> 10 Years	56.3% (9/16)	33.3% (50/150)		60.7% (17/28)	37.2% (51/137)		
Current smoker	12.5% (2/16)	17.3% (26/150)	1.00	10.7% (3/28)	17.5% (24/137)	0.58	1.00
Type 2 Diabetes Mellitus	6.3% (1/16)	3.3% (5/150)	0.46	17.9% (5/28)	2.9% (4/137)	0.008	0.39
eGFR (ml/min/1.73 m^2^)	85.8 ± 19.1	85.2 ± 15.7	0.88	82.3 ± 18.7	87.9 ± 16.8	0.12	0.55

Data presented as mean ± SD or % (*N*)

Comparison of baseline SBP and DBP for escape and non-escape patients is shown in Table 3. RDN patients who escaped had higher baseline 24 h SBP compared to non-escape RDN patients (156 ± 8 vs. 151 ± 8 mmHg, *p* = 0.010). Sham control patients who met escape criteria had higher baseline office SBP (167 ± 8 vs. 162 ± 7 mmHg, *p* = 0.002) and higher baseline 24 h SBP (154 ± 7 vs. 150 ± 8 mmHg, *p* = 0.010) compared to non-escape control patients.

**Table 3:** Baseline blood pressure measurements of escape vs. non-escape patients in RDN and sham control groups

	RDN			Sham control			Escape RDN vs. Sham *P*-value
Mean ± SD or %	Escape (*N* = 16)	Non-Escape (*N* = 150)	*P*-value	Escape (*N* = 28)	Non-Escape (*N* = 137)	*P*-value	
Office SBP (mmHg)	164 ± 7	163 ± 8	0.68	167 ± 8	162 ± 7	0.002	0.19
Office DBP (mmHg)	102 ± 9	101 ± 7	0.81	103 ± 9	102 ± 7	0.52	0.69
24 h SBP (mmHg)	156 ± 8	151 ± 8	0.010	154 ± 7	150 ± 8	0.010	0.38
24 h DBP (mmHg)	100 ± 10	98 ± 7	0.29	99 ± 10	99 ± 7	0.92	0.74

RDN and sham control escape patients had similar characteristics and blood pressure at baseline (Tables 2 and 3).

**Fig. 1:**
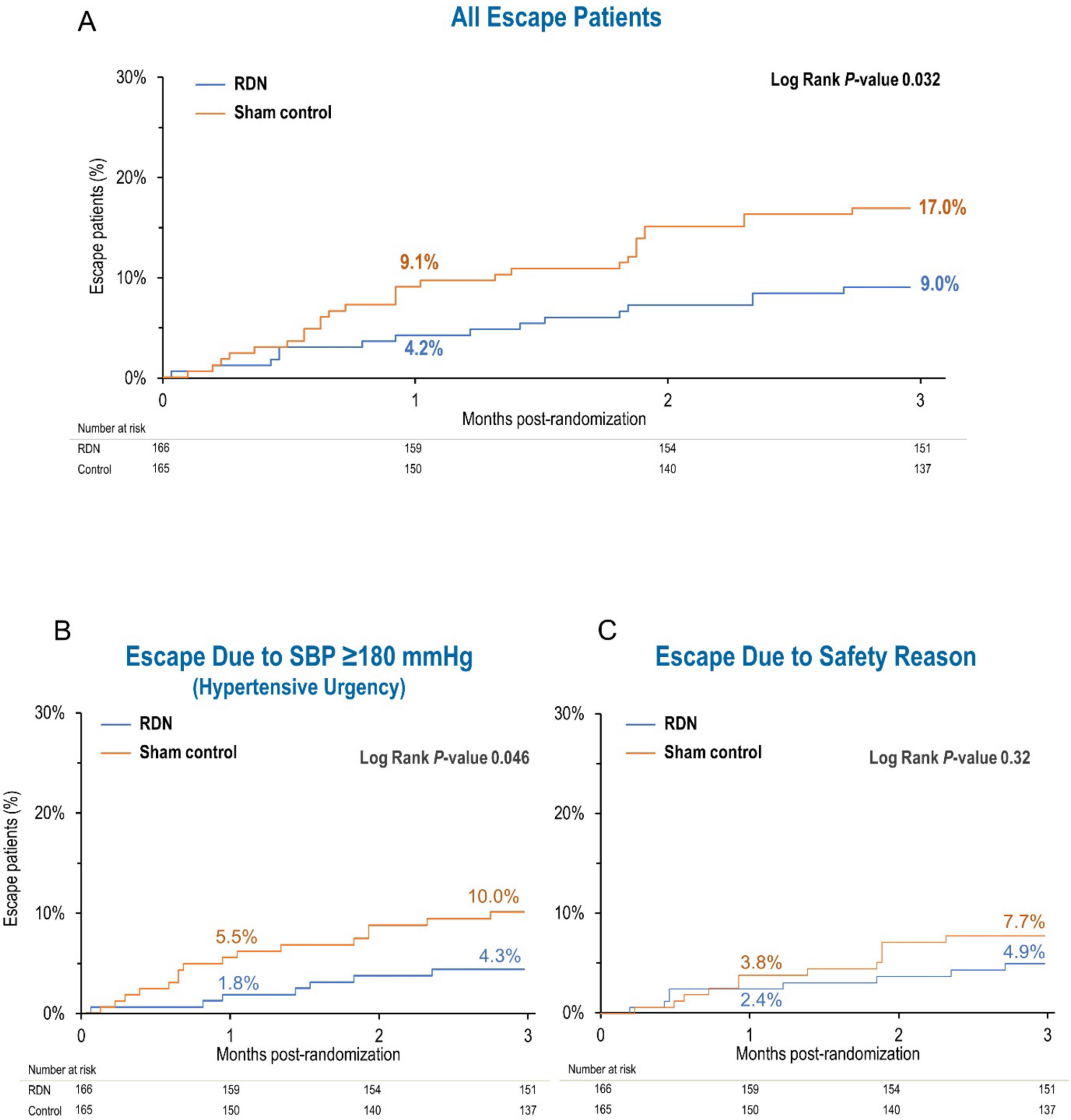
Kaplan–Meier estimate of rate of patients meeting escape criteria for RDN and sham control groups for **A** all escape patients, **B** patients with sustained office systolic BP ≥ 180 mmHg between randomization and 3 months, and **C** escape patients due to other safety concerns

Cumulative incidence curves with Kaplan–Meier estimates of rate of patients meeting escape criteria are shown in Fig. 1. There was a significantly higher rate of sham control patients meeting escape criteria compared to RDN patients for all escape patients (*p* = 0.032), as well as for patients with a potential hypertensive urgency with office SBP ≥ 180 mmHg (*p* = 0.046). Rate of escape was similar between RDN and sham control for patients who escaped for other safety reasons (*p* = 0.32).

## Discussion

The primary finding of this analysis was that in the SPYRAL HTN-OFF MED Pivotal trial, the sham control patient group met escape criteria as defined by a hypertensive urgency and other safety concerns more commonly than RDN patients throughout the primary 3 month follow up period (graphic abstract). Notably, since this analysis was performed in patients in the absence of anti-hypertensive medications, these results are not confounded by medication adherence issues. Patients with hypertensive emergencies or urgencies have a poor long-term prognosis [8] and reducing the frequency of these events could have important clinical impact. Potential clinical benefits of avoiding hypertensive emergency include reducing risk of an acute event such as a stroke, hospitalization, and also reducing the need for an acute BP intervention with additional clinic visits to assure an acceptable BP level.

Furthermore, for escape patients without 24 h SBP measurements prior to escape, the last observation carried forward 24 h SBP measurements were included in the primary endpoint analysis rather than BP measurements at time of escape. Nonetheless, 39 escape patients (including 15 in the RDN group and 24 in the sham control group) did not have an available 24 h SBP measurement prior to escape and thus did not contribute to the primary endpoint. This may have resulted in underestimation of the primary analysis of the treatment difference in 3 month 24 h SBP measurements since escape patients would likely have had relatively high 24 h SBP compared to non-escape patients.

This analysis highlights the relevance of RDN, since inhibiting sympathetic activity reduces BP variability [9] and thus minimizes the fluctuations that can cause BP to exceed critical levels associated with hypertensive urgency. A meta-analysis found BP variability to be associated with cardiovascular and mortality outcomes [10] and an “always on” therapy such as RDN [11] may provide a treatment option to reduce BP variability and cardiovascular events [12]. This inhibitory sympathetic effect may be of particular benefit in patients with type 2 diabetes in view of our finding that a disproportionate number of patients with diabetes in the sham-treated group—unlike the denervation group—required premature restoration of anti-hypertensive drug therapy.

Physician reasons for re-initiation of anti-hypertensive medications by physicians due to safety concerns are included in Table 1, but it is not clear whether all safety reasons were blood pressure-related. If such symptoms occurred at times remote from when blood pressures were actually measured a direct connection cannot be definitively concluded. A meta-analysis consisting of a pooled analysis of multiple RDN studies may be beneficial to better understand this question and further assess the effects of RDN on frequency of hypertensive urgencies.

While the primary endpoint of this trial was 3 months, Kaplan–Meier curves of rate of meeting escape criteria suggests RDN could have affected BP earlier. In a small study (73 patients), aortic stiffness was reduced 48 h after RDN, suggesting that this procedure can produce a relatively rapid response [13]. Pre-clinical studies may provide additional insight into timing of response to RDN.

## Limitations

The trial was not powered to assess differences in the rate of meeting escape criteria, and additional study is warranted. However, in this randomized trial consisting of 2 groups of patients with similar baseline characteristics, more patients in the sham control group met escape criteria compared to the RDN group. The decision to resume anti-hypertensive medication was based in part on physician discretion and therefore could have been biased. However, both clinicians and patients were effectively blinded to randomization [6], making potential bias less likely. Following the 3 month primary follow up period, all patients with uncontrolled BP initiated anti-hypertensive drug therapy and hence the escape criteria no longer applied. Therefore, it is unknown whether increased hypertensive urgency would have continued to grow after 3 months. However, the SPYRAL HTN-ON MED Pilot trial reported increased BP reduction at 6 months compared to 3 months follow up in the RDN group compared to sham control [11].

## Conclusions

In the SPYRAL HTN-OFF MED Pivotal trial, patients in the RDN group were less likely to experience hypertensive urgencies (SBP ≥ 180 mmHg) and other safety concerns that required immediate use of anti-hypertensive medications compared to the sham control group. This effect may be particularly relevant to patients with diabetes.
